# Computational Modeling Intervertebral Disc Pathophysiology: A Review

**DOI:** 10.3389/fphys.2021.750668

**Published:** 2022-01-13

**Authors:** Mallory Volz, Shady Elmasry, Alicia R. Jackson, Francesco Travascio

**Affiliations:** ^1^Department of Biomedical Engineering, University of Miami, Coral Gables, FL, United States; ^2^Department of Biomechanics, Hospital for Special Surgery, New York, NY, United States; ^3^Department of Mechanical and Aerospace Engineering, University of Miami, Coral Gables, FL, United States; ^4^Department of Orthopaedic Surgery, University of Miami, Miami, FL, United States; ^5^Max Biedermann Institute for Biomechanics, Mount Sinai Medical Center, Miami Beach, FL, United States

**Keywords:** disc degeneration, extracellular matrix, computational modeling, proteoglycans, homeostasis, aging, smoking, IGF-1

## Abstract

Lower back pain is a medical condition of epidemic proportion, and the degeneration of the intervertebral disc has been identified as a major contributor. The etiology of intervertebral disc (IVD) degeneration is multifactorial, depending on age, cell-mediated molecular degradation processes and genetics, which is accelerated by traumatic or gradual mechanical factors. The complexity of such intertwined biochemical and mechanical processes leading to degeneration makes it difficult to quantitatively identify cause–effect relationships through experiments. Computational modeling of the IVD is a powerful investigative tool since it offers the opportunity to vary, observe and isolate the effects of a wide range of phenomena involved in the degenerative process of discs. This review aims at discussing the main findings of finite element models of IVD pathophysiology with a special focus on the different factors contributing to physical changes typical of degenerative phenomena. Models presented are subdivided into those addressing role of nutritional supply, progressive biochemical alterations stemming from an imbalance between anabolic and catabolic processes, aging and those considering mechanical factors as the primary source that induces morphological change within the disc. Limitations of the current models, as well as opportunities for future computational modeling work are also discussed.

## Introduction

Lower back pain is a medical condition of epidemic proportion, costing over $100 billion annually in both direct and indirect expenditures in the United States alone. The nature of this condition is multifactorial, and symptomatic intervertebral disc (IVD) degeneration has been identified as a significant contributor (Kepler et al., 2013). The process of IVD degeneration occurs as a perturbation of the homeostatic balance between synthesis and degradation of its extracellular matrix (ECM) components: cell catabolic activity exceeds anabolism. As it progresses, IVD degeneration can cause structural failure, change in the composition and morphology of the ECM, calcification of the vertebral endplates, presence of osteophytes, cracks and fissures in the ECM, delamination and tears within the annulus fibrosus (AF), and collapse of the intervertebral space. Individuals undergoing this process experience pain and a progressive, irreversible loss of normal biomechanical function (Iatridis et al., 2013; Ito and Creemers, 2013; Urban and Fairbank, 2020). The etiology of IVD degeneration has not been fully delineated. However, it is believed that this process may result from an age-dependent, cell-mediated molecular degradation process under significant genetic influence that is induced and accelerated by traumatic or gradual mechanical factors, as well as mediated by toxic influences that trigger metabolic reactions (Baumgartner et al., 2021b).

Disc degeneration results from a number of preconditions and causal factors, both from internal and external impetuses, acting collectively. The individualized genetic makeup and morphology of a person’s IVDs can make them inherently more prone to degeneration with age. Biochemical changes that result from imbalances in disc metabolism also further degrade the disc. In addition, mechanical trauma, the severity of which depends on the associated force and time, exacerbates degeneration. This range of degenerative phenomena interact with each other and vary between individuals. The complexity of the degenerative process makes it difficult to quantitatively identify and measure the precise steps leading up to final mechanical failure. Since contributing factors are so intertwined with each other, traditional experimental models that focus on a single factor while controlling the others do not suffice. Therefore, alternative approaches that can enable controlling multiple factors simultaneously, such as computational modeling, was considered to elucidate the degeneration process.

**FIGURE 1:**
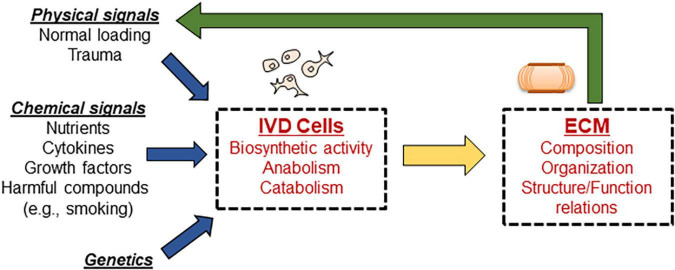
Schematic showing the complex interplay of events responsible for maintaining disc homeostasis. Disc cells are responsible for maintaining the ECM; any alteration to input signals guiding cellular biosynthesis results in altered disc ECM properties, which further alters signaling in a cyclic fashion. Computational modeling can be used to isolate the effects of individual signals, as well as investigate the combined influences.

Computational modeling is a powerful tool to study complex biological systems which is garnering more and more popularity in the scientific community. This methodological approach has also been adopted to investigate IVD degenerative changes. Computational models of IVD are based on *in vitro* and *in vivo* experimental measurements and offer the ability to vary and observe the effects of a wide range of intertwining phenomena involved in the degenerative process of discs, see Figure 1. Computational modeling has the advantage of being able to predict changes in the *in vivo* environment of the disc without completing the costly, time intensive and extremely difficult, if not impossible, task of measuring directly. Furthermore, computational analysis allows for more precise control and testing of individual parameters, which can lead to a better understanding the complex interplay of numerous factors in the disc.

This review aims to discuss the main findings of computational models of IVD pathophysiology with a special focus on the different factors contributing to physical changes typical of degenerative phenomena. It is acknowledged that computational modeling is a broad denomination including many methodological approaches (e.g., finite element analysis, multibody system modeling, agent based modeling, etc.). This review focuses on those computational models based on finite element analysis. Specifically, the models studied are subdivided in the following groups: (1) those investigating the role of nutrient supply on the viability and metabolism of disc cells; (2) those addressing progressive biochemical alterations stemming from an imbalance between anabolic and catabolic processes; (3) those addressing the natural process of aging as a contributor to IVD degradation; and (4) those considering mechanical factors as the primary source that induces morphological change within the disc.

It is noted that genetic factors are recognized as important contributors to the degenerative processes taking place in the IVD (Feng et al., 2016). However, to date, there are no computational models explicitly accounting for the role of genetics on the homeostasis of disc ECM. Therefore, discussion on genetic predisposition on the onset and progress of disc degeneration are excluded from this review.

## Nutritional Supply

Poor nutritional supply is believed to be a primary contributor to disc degeneration (Urban and Roberts, 2003; Hadjipavlou et al., 2008). Cells in the outer regions obtain nutrients from peripheral blood vessels and from limited capillary penetration into the disc (Hadjipavlou et al., 2008). However, the capillary network diminishes rapidly with age (Hadjipavlou et al., 2008). To reach cells in the inner disc regions, nutrients are transported by diffusion through the cartilage endplate (CEP) and into the IVD (Hadjipavlou et al., 2008). Changes in the diffusive transport abilities of the disc could also impact nutrient delivery. Importantly, the disc requires nutrients such as glucose and oxygen for cells to remain alive and active; it has been shown that cell density decreases linearly when glucose concentrations fall below 0.5 mM, and at concentrations below 0.2 mM or below, all disc cells become non-viable (Horner and Urban, 2001; Bibby et al., 2002; Bibby and Urban, 2004).

Computational models have been focusing on three areas of the nutrition pathways: (1) changes in CEP permeability to nutrients as a result of calcification, (2) cellular metabolic rates, and (3) disc geometry as a result of loading. Specifically, changes in CEP permeability to nutrients has been operationalized in several ways. In some cases, the boundary concentration of nutrients (both glucose and oxygen) at the CEP was reduced down to 50% (Kauppila, 1995; Selard et al., 2003; Malandrino et al., 2014), 30% (Zhu et al., 2012; Gu et al., 2014), or even 0% (Zhu et al., 2014, 2016, 2017) of the normal physiological values. Some other studies simulated the effect of CEP calcification by reducing the CEP exchange area with the nucleus pulposus (NP) and AF (Selard and Urban, 2003), or by reducing its water content (Soukane et al., 2007; Shirazi-Adl et al., 2010; Jackson et al., 2011a), thus reducing solute diffusivity into the core of the IVD tissue. In general, a reduction in the CEP permeability or exchange area led to decreased nutrient levels in the tissue, particularly moving farthest away from the nutrient supply. In cases where cell death was modeled, this was exacerbated by the limited nutrient exchange across the CEP. The effect of hindrance on solute transport due to loss of tissue water content was also directly investigated in Baldoni and Gu (2019). In that contribution, loss of water content was the result of depletion of fixed charge density, which is associated to the concentration of glycosaminoglycans (GAGs) present in the ECM of the IVD. Interestingly, a study including reduced CEP exchange area as well as reduction in tissue water content in each region of the disc to reflect degeneration, found that nutrient distribution in the IVD depends primarily on tissue porosity (which is related to solute diffusivity) and CEP exchange area, and very little on the elastic modulus or fixed charge density of the tissue (Magnier et al., 2009). It should be noted that the process of CEP calcification occurs toward the late stages of disc degeneration (Benneker et al., 2005a,b). Since CEP undergoes compositional changes as degeneration progresses (Fields et al., 2015), a finite element analysis was carried out to appraise the effects of such changes on disc nutrition and cell viability (Ruiz et al., 2018). The model predicted that CEP permeability increases with the level of degeneration, in agreement with some experimental measurement in the literature (Williamson et al., 2001), and that CEP degeneration might be directly related to the dehydration of the NP. It was also found that CEP degeneration implicated cell starvation and death. This theoretical suggests a path for the early stages of disc degeneration.

The effect of cellular metabolic rates on nutrient levels in the disc have also been investigated. Selard and co-workers directly addressed the effect of changes in cell metabolism on disc health by simulating cellular consumption–production rates varying from 50 to 200% of the normal physiological values (Selard and Urban, 2003). Magnier and co-workers investigated effects of altered cell density, which directly effects the overall consumption of nutrients in the tissue (Magnier et al., 2009). In general, these studies found that higher consumption rates led to a depletion of nutrients in the disc. Physiologically, this reflects the nutrient limitations in the IVD and the role of nutrition in the degeneration cascade; further, these studies have important implications for treatment strategies for disc degeneration, particularly those involving injections of cells into the disc. The effect of changes in disc height as a result of normal physiological loading conditions was investigated by simulating mechanical loads typical of supine, standing, and weight-bearing standing conditions in both normal and degenerated disc (Jackson et al., 2011b). It was found that, compared to the supine position, standing and weight-bearing caused a change in tissue water content which led to decreased glucose levels throughout the disc. For the degenerated disc, the minimum glucose levels were below threshold values for cellular survival. Also, the effect of sustained compression on oxygen metabolism in the IVD was investigated *via* poromechanical finite element model of the human disc coupled with oxygen and lactate transport (Malandrino et al., 2011). It was found that external loads affect both oxygen and lactate regional distributions within the disc, with larger effects observed in healthy tissue when compared to degenerated case. This would suggest that healthy disc properties have a positive effect of loading on metabolic transport and remark the link existing between disc function and nutrition.

## Biochemical Alterations

### Growth Factors as Modulators of Disc Anabolism

Growth factors contribute to the upregulation of IVD cellular anabolism by increasing proteoglycan synthesis and cell proliferation (Masuda and An, 2004). Accordingly, maintaining normal levels of these signaling proteins is important for disc health (Masuda and An, 2004; An et al., 2006). The role of growth factors in disc homeostasis involves many coupled phenomena, which makes it hard to elucidate exclusively by experimental studies. Therefore, computational models have been developed in order to shed light on their effects on the health of the IVD. While several growth factors are known to play key roles in disc homeostasis [e.g., basic fibroblast growth factor (bFGF), transforming growth factor (TGF), insulin-like growth factors (IGFs), etc.] (Hadjipavlou et al., 2008), to our best knowledge, computational models have only investigated the signal pathways of IGF-1 (Travascio et al., 2014; Asfour et al., 2015; Elmasry et al., 2015, 2016).

**FIGURE 2:**
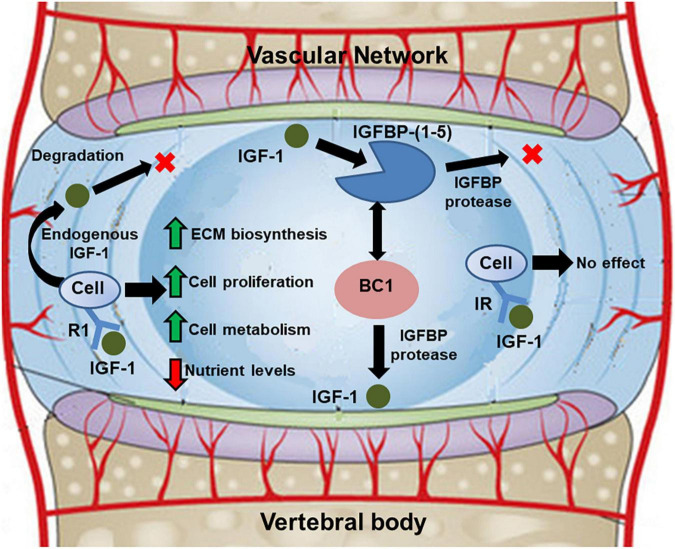
Schematic showing diffusive-reactive transport of IGF-1 in the disc, and its implications on cell metabolism and biosynthetic activity. Adapted from Elmasry et al. (2016).

The theoretical frameworks used to describe the mechanism of IGF-1 signaling in the disc evolved from the theoretical models for cartilage homeostasis proposed by Zhang and co-workers (Zhang et al., 2009, 2010, 2013; see Figure 2). The growth factor was assumed to be delivered exogenously from the surrounding vascular network or endogenously secreted by disc cells. Within the ECM, the IGF-1 would either bind to IGF-specific cell surface receptors or combine, reversibly, with IGF-binding proteins (Travascio et al., 2014). Binding to cell surface receptors would enhance cell proliferation and synthesis of GAGs in a dose-dependent fashion, as reported in *in vitro* studies (Osada et al., 1996; Pratsinis and Kletsas, 2007). Given its anabolic potential, computational studies have evaluated the effect of exogenous administration of IGF-1 to boost ECM biosynthesis in degenerated discs (Huang et al., 2012; Travascio et al., 2014; Elmasry et al., 2016). Intradiscal injections of the growth factor (Huang et al., 2012) and systemic delivery (Travascio et al., 2014; Elmasry et al., 2016) were simulated. In both cases, the exogenous administration of IGF-1 caused an initial increase in GAG synthesis, followed by a dramatic drop in cell density within the tissue: increase in IGF-1 causes an initial increase in cell proliferation rate, as well as cell metabolism. Such an increase becomes unsustainable unless delivery of nutrients is increased as well. Overall, these studies suggested that exogenous administration of IGF-1 alone is not a viable option to restore the integrity of the ECM of a degenerated IVD.

Several pro-inflammatory signals upregulating IVD cell catabolism and/or apoptosis have been individuated (e.g., TNF-α, metalloproteinases, interleukins, etc.) (Bisson et al., 2021). However, to our best knowledge, there are no computational models specifically investigating the role of these cytokines in the disc, with the exception of the recent work from Baumgartner et al. (2021a): using an agent based approach, the authors developed and validated a model capable of calculating mRNA expression of aggrecan, collagen type I, collagen type II, MMP-3, and ADAMTS for inflamed and non-inflamed cells. This pioneering model may have the potential to provide mechanistic interpretation on the cascade of effects leading to disc degeneration upon inflammation.

### Detrimental Effects of Smoking

**FIGURE 3:**
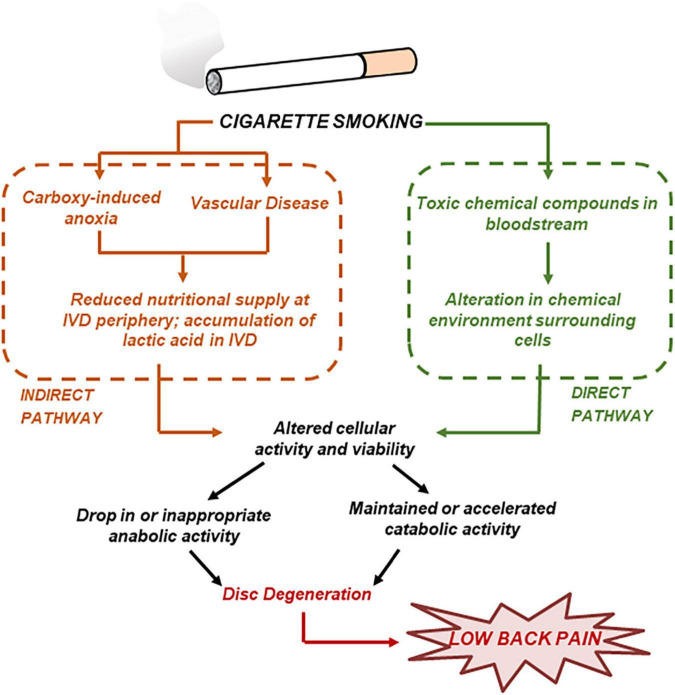
Cigarette smoking may result in disc degeneration by both direct and indirect pathways. Adapted from Jackson et al. (2015).

Several studies investigated the detrimental effect of smoking on the health of the IVD (Jackson et al., 2015). Most importantly, animal studies showed that smoking causes a build-up of carbon monoxide, which binds to hemoglobin to displace oxygen and cause anoxia; this, in addition to related vascular constriction, leads to a reduced nutritional supply in the disc (Holm and Nachemson, 1988). Moreover, *in vitro* studies have shown that nicotine reduces cell proliferation and ECM biosynthesis in a dose-dependent fashion (Akmal et al., 2004). Based on the wealth of information provided by experimental studies, a system of “direct” and “indirect” pathways of smoking to disc degeneration has been proposed (Jackson et al., 2015; see Figure 3). A computational model including both the “direct” and “indirect” pathways of smoking was developed to quantify the effect of tobacco smoking on the homeostasis of disc degeneration (Elmasry et al., 2015). Both “moderate” and “heavy” smoking scenarios were investigated. It was found that “heavy” smoking significantly reduced cell viability in most of the NP region of the disc. Most importantly, it was found that the damages induced from “heavy” smoking were permanent in that, upon cessation of smoking, the cell population within the NP would only recover to 75% of its normal physiological value (Elmasry et al., 2015).

## Effect of Aging

In the disc, senescent cells gradually lose their ability to proliferate (Adams and Roughley, 2006; Hadjipavlou et al., 2008; Kepler et al., 2013), so that their density declines, most notably in the annulus (Hadjipavlou et al., 2008). It is also known that the rate of synthesis of proteoglycans decreases with age (Adams and Roughley, 2006; Hadjipavlou et al., 2008). Moreover, the proteoglycans that continue to be produced are smaller and less aggregated because of a decline in link proteins and type IX collagen (Kepler et al., 2013). Furthermore, the collagen of the nucleus transitions from type II to a more fibrous type I (Adams and Roughley, 2006; Hadjipavlou et al., 2008; Kepler et al., 2013), thereby weakening the structural integrity of the nucleus and causing the distinction between the nucleus and annulus to become less and less apparent with advancing degeneration (Urban and Roberts, 2003; Hadjipavlou et al., 2008; Kepler et al., 2013). Zhu and co-workers (Zhu et al., 2012; Gu et al., 2014) provided a comprehensive model of disc degeneration that analyzed the changes in cell density, nutrition level, proteoglycans (PGs) and water contents, and volume change in the disc over the progression of degeneration across decades. Their model showed that changes in cell density equilibrated within 30 days following reduction in nutrient supply, while tissue water and PG content took 55 years to reach a new equilibrium. Their results were consistent with disc composition measurements in the literature. Asfour and co-workers directly simulated the effect of aging on cell metabolism (Asfour et al., 2015), whose experimental evidence was based on *in vitro* studies (Okuda et al., 2001). It has been observed that senescent disc cells reduced the production of endogenous IGF-1, reduced the expression of IGF-specific cell surface receptors, and increased the production of IGF-binding proteins. Computer simulations showed that while reduction of endogenous production of IGF-1 and expression of IGF surface receptors had a detrimental effect on the anabolism of disc cells, the increase in expression of IGF-binding proteins helped mitigate the detrimental effects of aging on disc homeostasis: by binding to IGF-1, these proteins extended the lifetime of the growth factor within the ECM of the disc, thus allowing for more opportunities for signaling with the cell surface receptors. It was hypothesized that the increase of IGF-binding protein was a mechanism of defense of the disc cells against aging.

## Mechanical Factors

Abnormal mechanical loads and motion patterns significantly contribute to the onset and progression of degenerative changes in the disc (Urban and Roberts, 2003; Stokes et al., 2011; Bashkuev et al., 2018). Onset of degeneration may be from a single injury or from wear-and-tear fatigue (Adams and Roughley, 2006). Degeneration is a cascading event that is often attributed to the cumulative damage caused by various combinations of static and quasi-static loads, and vibrations (An et al., 2006; Natarajan et al., 2006; Woo et al., 2007). The changing structure and mechanical performance of the degenerating disc increasingly affects its ability to support and transfer loads (Adams and Roughley, 2006; Ruberte et al., 2009). Hence, degeneration often spreads to adjacent disc levels due to altered load sharing between spine units (Adams and Roughley, 2006; Ruberte et al., 2009; Bashkuev et al., 2018).

There is great variability in types and magnitudes of spinal loads among individuals. Computational modeling can be highly valuable in investigating how singular and combined loading conditions may contribute to degeneration.

### Static and Quasi-Static Loading

Excessive static compressive loading causes abnormally high stresses and strains to develop within the disc, leading to altered biologic activity, cell death and tissue rearranging, and diminished mechanical performance (Setton and Chen, 2006). Over time this can lead to tissue degeneration and failure. Lotz and co-workers used FE modeling to depict both the onset and progression of degeneration in discs as a result of static loading (Lotz et al., 1998). As the magnitude of the compression increases and degeneration progresses, the stress in the annulus moves from the inner surface to the outer surface (Lotz et al., 1998).

Torsion coupled with compression has been related to degenerative changes in the disc. Weighted torsion has been linked to lower back pain in individuals who often perform the lift-and-twist motion (Marras et al., 1995; Lötters et al., 2003). Exposure to weighted torsion alters the disc composition. Disc fibers become damaged when elongated past their resting length (Hadjipavlou et al., 2008). The extent of strain and thus damage is highly dependent on the angle of rotation, which is normally no greater than 9° (Farfan et al., 1970). The effects of axial rotation, flexion and extension, and lateral bending on the disc are the motion patterns most commonly simulated in computational models. A model by Ibarz et al. (2013) showed that the degree of instability of a lumbar disc segment was dependent on the extent of degeneration of the discs. For simulated degenerated conditions, higher mobility was detected in a range of motions at every vertebral level of the lumbar spine, compared to a healthy control (Ibarz et al., 2013). This relationship is likely due to the decrease in pressure and swelling ability of the NP, the main load-bearing mechanism of the disc, with degeneration: load mechanics are altered due to disc material disruptions, and the spinal segment becomes increasingly unstable (Adams and Roughley, 2006).

### Vibrations

Mechanical vibrations have been associated with disc degeneration, and it has been suggested that they exert their greatest impact on the disc when they match the resonant frequency of the spine (Wilder et al., 1982; Panjabi et al., 1986; Pope et al., 1991). Epidemiological studies have shown that drivers of trucks, buses, and tractors experience high rates of back pain and herniation, likely due to their exposure to whole body vibrations within the resonant range of the spine (Kelsey, 1975a,b; Kelsey and Hardy, 1975; Frymoyer et al., 1980; Heliövaara, 1987; Bongers et al., 1990; Bovenzi and Zadini, 1992). Models investigating the mechanical effects of sinusoidal loading at different vibrational frequencies on the discs were developed. The range of frequencies explored varied between 1 and 11 Hz (Guo and Teo, 2006; Fan et al., 2020), to simulate those mechanical loading conditions typical of vehicle driving. Simulations included the cases of healthy, and moderately and severely degenerated discs. It was found that vibrations caused decrease in the fluid pressure and increase in the effective stress in the AF, with larger effects observed in degenerated discs, when compared to healthy ones (Guo and Teo, 2006; Fan et al., 2020). Overall, these results indicate that already degenerated discs experience accelerated rates of degradation, when compared to healthy discs, when exposed to vibrations.

### Effects of Shape, Size and Structural Changes, and Implications on Other Disc Levels

The effects of variations in disc axial area, depth, width, and height on tissue degeneration were investigated *via* computational modeling (Peloquin et al., 2014). It was observed that only disc area significantly correlated with degeneration. However, it is unknown as to whether degeneration causes the disc to bulge and increase in area, or if larger discs are simply more prone to degeneration (Peloquin et al., 2014). In any case, this study supports the understanding that variations in disc geometry should be included in models to obtain the most generalizable results when analyzing the mechanical behavior of the spine. Also, annular lesions have been associated with onset and progression of degeneration. The effects of rim, radial, and circumferential annular lesions on disc mechanics were investigated *via* computational modeling. However, the results suggested that lesions alone have minimal impact on the overall mechanical behavior of the disc (Little et al., 2007).

Previous experimental studies have shown that discs adjacent to degenerated discs are significantly more likely to experience degeneration (Elfering et al., 2002; Waris et al., 2007). To investigate the etiology of the adjacent IVD degeneration, computational models have artificially induced degeneration at one-disc segment and observe how the biomechanics of normal, adjacent segments were affected (Kim et al., 1991; Ruberte et al., 2009; Hussain et al., 2010, 2012). Abnormal biomechanics in the adjacent disc levels was observed. In particular, intradiscal pressure and disc bulge in the adjacent discs increased, and segment motions were significantly altered. All these changes suggest possible mechanisms by which degeneration spreads from one level to the adjacent ones. Computational models investigating adjacent segment disease secondary to spine surgery have also been presented. Partly based on a previously validated computational model of a spine segment incorporating a biochemical composition-based model of the IVD (Barthelemy et al., 2016), van Rijsbergen et al. (2018) further developed a coupled and patient-specific mechano-regulated model to predict disc generation and changes in bone density after spinal fusion (van Rijsbergen et al., 2018). The model was successfully validated with clinical follow-up data, and constitutes a powerful tool to understand the effect of surgical intervention on the adjacent tissue remodeling (both bone and IVD).

## Limitations of the Models and Future Directions

Computational modeling is a valuable tool that can be harnessed to aid in better understanding of IVD degeneration, as well as for designing and validating novel strategies to treat and/or prevent disease progression. However, it is important to note that the accuracy of theoretical prediction is founded on robust model inputs. As such, the primary limitation hindering model development is a lack of quantitative data available for building computational models. For instance, most relationships for IVD cellular activity are based on *in vitro* studies primarily in animal models, which likely do not represent the *in vivo* human disc environment. Furthermore, a lack of empirical data may also lead to a simplification of the disc computational model. In metabolic studies, the theoretical frameworks do not account for the multitude of factors that may affect cellular activity or relevant coupling/interaction effects, which could significantly alter model prediction. In mechanical studies, complex muscle interactions, the viscoelastic behavior of disc fibers and ligaments, and dynamic material properties are often discounted in the computational model, which also can influence the model accuracy.

Despite these limitations, there remains a great deal of progress that can be made to improve and implement computational modeling of the IVD. Modelers must work hand-in-hand with those at the benchtop to produce and incorporate robust quantitative data into computational models, to improve the prediction accuracy. Moreover, effort must be focused on accomplishing strong model validation, to ensure that model prediction matches the *in vivo* environment in the IVD. However, because of the difficulty in obtaining *in vivo* data in the IVD (Wilke et al., 1999), models are often developed and successfully validated based on *ex vivo* data. For instance, Noailly et al. (2007) compared theoretical predictions for spinal range of motion with cadaveric measurements to validate their computational model. Similarly, Zhu et al. (2017) also used experimental results for disc water content to validate their computational prediction of disc degeneration. Recently, bioreactor studies have been used to validate finite elements models (Gatenbein et al., 2015). For instance, data obtained from *ex vivo* loading of goat discs in a bioreactor (Paul et al., 2013, 2017) were used to validate a model predicting disc height variation upon chondroitinase ABC injection (Castro et al., 2014). Finally, with the current thrust for personized medicine, individualized computational modeling, which may incorporate patient-specific geometry and/or tissue properties based on imaging, has a great potential to improve treatment strategies and patient outcomes for disc degeneration. Subject-specific finite element modeling can provide valuable quantitative biomechanical information to clinicians through non-invasive, time, and cost effective means (Nikkhoo et al., 2019). Since traditional methods of investigating IVD degeneration are difficult to measure directly and often do not represent the human disc, personalized modeling could be the solution for planning surgery or prescribing biotherapies for disc degeneration (Hu et al., 2019; Molinari and Falcinelli, 2021).

## Author Contributions

All authors listed have made a substantial, direct, and intellectual contribution to the work, and approved it for publication.

## Conflict of Interest

The authors declare that the research was conducted in the absence of any commercial or financial relationships that could be construed as a potential conflict of interest.

## Publisher’s Note

All claims expressed in this article are solely those of the authors and do not necessarily represent those of their affiliated organizations, or those of the publisher, the editors and the reviewers. Any product that may be evaluated in this article, or claim that may be made by its manufacturer, is not guaranteed or endorsed by the publisher.
